# Etiology of autistic features: the persisting neurotoxic effects of propionic acid

**DOI:** 10.1186/1742-2094-9-74

**Published:** 2012-04-24

**Authors:** Afaf K El-Ansary, Abir Ben Bacha, Malak Kotb

**Affiliations:** 1 Biochemistry Department, Science College, King Saud University, Riyadh, Saudi Arabia; 2 Department of Molecular Genetics, Biochemistry and Microbiology, College of Medicine, University of Cincinnati, Cincinnati, Ohio, USA

## Abstract

**Background:**

Recent clinical observations suggest that certain gut and dietary factors may transiently worsen symptoms in autism. Propionic acid (PA) is a short chain fatty acid and an important intermediate of cellular metabolism. Although PA has several beneficial biological effects, its accumulation is neurotoxic.

**Methods:**

Two groups of young Western albino male rats weighing about 45 to 60 grams (approximately 21 days old) were used in the present study. The first group consisted of oral buffered PA-treated rats that were given a neurotoxic dose of 250 mg/kg body weight/day for three days, n = eight; the second group of rats were given only phosphate buffered saline and used as a control. Biochemical parameters representing oxidative stress, energy metabolism, neuroinflammation, neurotransmission, and apoptosis were investigated in brain homogenates of both groups.

**Results:**

Biochemical analyses of brain homogenates from PA-treated rats showed an increase in oxidative stress markers (for example, lipid peroxidation), coupled with a decrease in glutathione (GSH) and glutathione peroxidase (GPX) and catalase activities. Impaired energy metabolism was ascertained through the decrease of lactate dehydrogenase and activation of creatine kinase (CK). Elevated IL-6, TNFα, IFNγ and heat shock protein 70 (HSP70) confirmed the neuroinflammatory effect of PA. Moreover, elevation of caspase3 and DNA fragmentation proved the pro-apoptotic and neurotoxic effect of PA to rat pups

**Conclusion:**

By comparing the results obtained with those from animal models of autism or with clinical data on the biochemical profile of autistic patients, this study showed that the neurotoxicity of PA as an environmental factor could play a central role in the etiology of autistic biochemical features.

## Introduction

Autism spectrum disorders (ASDs) are a cluster of related neurodevelopmental disorders characterized by varying degrees of impaired socialization, reduced communication, and limited, repetitive, or stereotyped interests and activities. Patients with ASD are often reported to suffer from a variety of bowel dysfunctions and gastrointestinal disturbances [1]. Current estimates indicate that the prevalence of autism has increased by more than 10-fold in the last three decades.

The lack of a full understanding of the relationship between the brain and the behavior of autistic patients has hindered the development of effective methods for the diagnosis and management of autism. In fact, a significant limiting factor in understanding this relationship lies in the difficulties associated with obtaining human brain tissues from both normal subjects and sample patients [2].

Various animal models have been employed to investigate the environmental factors, core symptoms, possible causes, and potential treatments of neurodevelopmental disorders. Among the several animal models so far assayed, the rat model appears to be an excellent standard experimental system, namely because of the ample data already available on the genetics and behavioral phenotyping of various rat strains [3].

There has been a growing interest in the literature on possible environmental agents involved in the development of autism, such as chemical toxins, which could act during critical periods of pre- and early postnatal development [4].

Of particular relevance to this argument, propionic acid (PA) has often been reported to induce a number of behavioral changes and neuroinflammatory responses in rats reminiscent of ASD. This dietary short chain fatty acid is a common food preservative and metabolic end-product of enteric bacteria in the gut. Although mostly accumulating in the gut, PA can readily cross the gut-blood and blood–brain barriers (BBB) and gain access to the central nervous system (CNS). In the brain, it can cross cell membranes and accumulate within cells, inducing intracellular acidification [5,6], which may alter neurotransmitter releases and, ultimately, neuronal communication and behavior [7,8].

MacFabe et al. [9] showed that intraventricular infusion of PA can change both brain and behavior in the laboratory rat in a manner that is consistent with symptoms of human ASD. The behavioral, neuropathological and biochemical findings in the MacFabe PA model provide further support for the hypothesis that autism may be a systemic metabolic encephalopathic process affecting the brain. They have also found evidence of reversible impairments in social behavior following PA exposure [10]. The similarities in innate neuroinflammatory and oxidative stress changes between their animal model and human ASD cases could represent similar metabolic or immune-mediated processes [11] directly or indirectly associated with PA. Of particular interest are their observations of broad impairments in glutathione (GSH) and catalase metabolism which could provide a common mechanism for increased oxidative stress and increased environmental sensitivity to a variety of environmental compounds [12].

Moreover, there are a series of inherited and acquired conditions which lead to elevations of PA and other short chain fatty acids and these are related to developmental delay, seizure disorder and gastrointestinal symptoms, resembling some aspects of autism [13,14]. Thus, PA may be a putative link between dietary or enterobacterially derived metabolites along with genetic predisposition and subsequent features of autism.

Considering the serious concerns expressed over the alarming rates with which ASDs are increasing throughout the world and in light of the promising opportunities that PA administration might bring with regard to the understanding of these neurodevelopmental disorders, the present study was undertaken to investigate and evaluate the neurotoxic effects associated with the oral administration of PA to rats. PA was chosen for this study because it has the lowest Km (2.03), higher affinity to transporters and highest brain uptake index (43.53%) compared to other short chain fatty acids related to neurodevelopmental disorders. Moreover, propionate was the only short chain fatty acid which had a lower plasma concentration and high rate of influx to the brain in autistic patients compared to healthy age-matched controls [15,16]. Oral administration of PA was selected in an attempt to understand the gut-brain axis hypothesis in PA neurotoxicity and to find biochemical correlates to different autistic features with reference to data previously reported either for the MacFabe animal model of autism or clinical findings for children affected with autism. Accordingly, several biochemical parameters, related to oxidative stress/antioxidant status, energy metabolism, neuroinflammation, neurotransmission and apoptosis were selected for measurement and analysis.

## Material and methods

### Animals

The experimental assays for this study were performed on 16 young (approximately 21 days old) male western albino rats (45 to 60 g). Rats were obtained from the animal house of the pharmacy college, King Saud University, and were randomly assigned to two groups of eight rats each. The first group of rats were given an oral neurotoxic dose of PA (250 mg/kg body weight/day for three days; n = eight) [17] and were termed the oral buffered PA-treated group. The second group consisted of rats to which only phosphate buffered saline was administered and were used as a control group (n = eight). The two groups of rats were individually housed under controlled temperature (21 ± 1°C) with *ad libitum* access to food and water. The protocol of the present work was approved by the Ethics Committee at the King Saud University and all experiments were performed in accordance with the guidelines of the National Animal Care and Use Committee.

### Tissue preparation

At the end of the feeding trials, the rats were anesthetized with carbon dioxide and decapitated. The brain was removed from the skull and was dissected into small pieces and homogenized as a whole in 10 times w/v bi-distilled water and kept at −80°C until further use for different biochemical analyses.

### Biochemical analyses

#### Measurement of lipid peroxidation

Lipid oxidation was evaluated by measuring the levels of lipid peroxidation by-products as thiobarbituric acid reactive substances (TBARS), namely malondialdehyde (MD), using the method of Ruiz-Larrea et al. [18]. Accordingly, the samples were heated with TBA at low pH and the formation of a pink chromogen was measured by absorbance at 532 nm. The concentration of lipid peroxides was calculated as μmoles/ml using the extinction coefficient of MD.

#### Assay of vitamin C

Assay of vitamin C was performed according to the method of Jagota and Dani [19]. A quantity of 0.2 ml of brain homogenates was mixed with 0.8 ml of 10% trichloroacetic acid (TCA) and incubated in ice for 5 minutes. The samples were then centrifuged for 10 minutes at 3,500 rpm and 4°C. An amount of 1.5 ml double distilled water was subsequently added to 0.5 ml of the supernatant. Eventually, 2 ml of Folin-phenol reagent were added and absorbance was measured at 760 nm after 10 minutes.

#### Assay of glutathione (GSH)

GSH content was determined according to the method described by Beutler et al. [20] using 5,5′-dithiobis 2-nitrobenzoic acid (DTNB) with sulfhydryl compounds to produce a relatively stable yellow color.

#### Assay of glutathione peroxidase (GPX)

GPX was assayed according to the method of Paglia and Valentine [21]. In this assay, cumene hydroperoxide is used as the peroxide substrate (ROOH) and glutathione reductase (GSSG-R) and NADPH (β − nicotinamide adenine dinucleotide phosphate, reduced) are included in the reaction mixture. The formation of oxidized glutathione (GSSG) catalyzed by GPX is coupled to the recycling of GSSG back to GSH using GSSG-R. NADPH is oxidized to NADP^+^. The change in absorbance at 340 nm due to NADPH oxidation is monitored and is indicative of GPX activity.

#### Assay of catalase

The total volume of the reaction mixture was 3 mL. It contained 1.5 mL of 0.2 M sodium phosphate buffer pH 7.2, 1.2 mL of 0.5 mM hydrogen peroxide and enzyme. The reaction was started by adding hydrogen peroxide (H_2_O_2_), and the rate of change in absorbance was measured at 240 nm for two minutes [22].Values were expressed as μmoles of H_2_O_2_ dissociated/minute/dL brain homogenates, that is, U/dL.

#### Assay of CK

CK was determined using the CK NSC kit for its simplicity as a product of NSC Human, Germany [23].

#### Assay of lactate dehydrogenase (LDH)

The quantitative determination of LDH in the brain homogenates was performed using the lactate-to-pyruvate kinetic method described by Henry et al. [24].

#### Assay of TNFα

TNF*α* was measured using a mouse TNF*α* ELISA kit (Hycult Biotech, Uden, Netherlands. The antibody reacts with the natural TNF*α* of the rats and identifies membrane as well as receptor-bound TNF*α*. The TNF*α* trimer interacts with either of the two types of TNF-R, leading to receptor cross-linking. One unit of Hycult Biotech Mouse TNF*α* approximates the bioactivity of 16 units of human TNF*α* according to the standard L929 cytotoxicity assays for TNF*α* prepared by the World Health Organization (WHO).

#### Assay of Caspase3

Caspase3 was measured using an ELISA kit, a product of Cusabio (Cusabio, Wuhan, China). The microtiter plate provided in this kit was pre-coated with an antibody specific for caspase3. Standards or samples were then added to the appropriate microtiter plate wells with a biotin-conjugated antibody preparation specific for caspase3. After that, avidin conjugated to horseradish peroxidase (HRP) was added to each microplate well and incubated. A TMB (3,3',5,5' tetramethyl-benzidine) substrate solution was then added to each well. Only the wells that contained caspase3, biotin-conjugated antibody, and enzyme-conjugated avidin would exhibit a change in color. The enzyme-substrate reaction was terminated by the addition of a sulfuric acid solution and color change was measured spectrophotometrically at a wavelength of 450 nm ± 2 nm. The concentration of caspase3 in the samples was then determined by comparing the optical density (O.D.) of the samples to the standard curve.

#### Assay of IL-6

IL-6 was assayed using a Quantikine ELISA kit (R & D Systems, Minneapolis, MN, USA. A microplate was pre-coated with a monoclonal antibody specific for rat IL-6. Fifty microliters (50μL) of each standard, control, or sample were placed in separate wells. The reagent was mixed by gently tapping the plate frame for one minute after being covered with the adhesive strip provided. The plate was incubated for two hours at room temperature and any rat IL-6 present was bound by the immobilized antibody.

After washing away unbound substances, an enzyme-linked polyclonal antibody specific for rat IL-6 was added to the wells. Following a subsequent wash step to remove unbound antibody-enzyme reagents, 100 μL of substrate solution was added to each well and the plate was incubated for 30 minutes at room temperature. The enzyme reaction yielded a blue product that turned yellow when the stop solution was added. The intensity measured for the color was in proportion to the amount of rat IL-6 bound in the initial step. The sample values were then read off the standard curve.

#### Assay of gamma amino-butyric acid (GABA)

GABA was quantitatively determined using the ELISA immunoassay kit from ALPCO Diagnostics (Salem, NH, USA). Volumes of 300 μL of diluted standards, controls and undiluted samples were placed into the appropriate wells of the extraction plate. A total of 300 μL of the diluent was added to each well. The wells were then covered with adhesive foils and shaken for 30 minutes at room temperature (20 to 25°C) on a shaker (600 rpm). Two washing cycles were performed, after which 250 μL elution buffers were introduced into the appropriate wells of the extraction plate, covered, and then shaken. A total of 100 μL of the extract was then used for the subsequent derivatization procedure. An amount of 10 μL of NaOH was added to each well, which was followed by the addition of 50 μL of the equalizing reagent (freshly prepared before the assay). The wells were then shaken for one minute on a shaker set at 600 rpm. A volume of 10 μL of the D-reagent was added to each well, which was then incubated for two hours (20 to 25°C). An amount of 150 μL of the Q-buffer was then added to the wells, which were incubated for ten minutes at room temperature (20 to 25°C) on a shaker (approximately 600 rpm). Volumes of 25 μL of the derivatives were then used for subsequent ELISA assays.

#### Assay of serotonin

Serotonin was measured using an ELISA kit from Immuno-Biological Laboratories (IBL, Hamburg, Germany). Brain homogenate preparation (derivatization of serotonin to N-acylserotonin) was part of the sample dilution and was achieved by the incubation of the respective sample with the acylation reagent. The assay procedure followed the competitive ELISA protocols whereby competition takes place between biotinylated and non-biotinylated antigen for a fixed number of antibody binding sites. The amount of biotinylated antigens bound to the antibody was inversely proportional to the N-acylserotonin concentration of the sample. When the system was in equilibrium, the free biotinylated antigens were removed by a washing step. The antibody-bound biotinylated antigens were determined using anti-biotin alkaline phosphatase as a marker and p-nitrophenyl phosphate as a substrate. The unknown samples were quantified by comparing enzymatic activity with reference to a response curve for known standards.

#### Dopamine assay

Dopamine was extracted by using a cis-diol-specific affinity gel, acylated and then derivatized enzymatically. Quantitavive assay was performed using an ELISA kit, a product of Immuno Biological Laboratories (IBL).

#### Assay of IFNγ

IFN*γ* was measured using an ELISA kit, a product of Thermo Scientific (Rockford, IL, USA) according to the manufacturer’s instructions. This assay employs a quantitative sandwich enzyme immunoassay technique that measures IFN*γ* in less than five hours. A polyclonal antibody specific for human IFN*γ* has been pre-coated onto a 96-well microplate with removable strips. IFN*γ* in standards and samples is sandwiched by the immobilized antibody and biotinylated polyclonal antibody specific for IFN*γ*, which is recognized by a streptavidin-peroxidase conjugate. All unbound material is then washed away and a peroxidase enzyme substrate is added. The color development is stopped and the intensity of the color is measured at 550 nm and subtracted from absorbance at 450 nm. The minimum level of rat IFNγ detected by this product is less than 2 pg/ml.

#### Phospholipids measurement

Briefly, 50 μl of brain homogenate was diluted with 750 μl deionized water followed by 2 ml of methanol and 1 ml of chloroform. The mixture was stirred (Rotary mixer 34526, Snijders) for 15 minutes and centrifuged for five minutes at 4,000 rpm [25]. Phospholipid separation was performed using a Kaneur Maxi Star HPLC system with four solvent lines and a degasser SEDEX 55 evaporating light detector (SEDEX 55 Lichtstreu detector, S.E.D.E.E., Sedere, Alfortville, France) coupled with Apex M625 software (Autochrom, Milford, HA, USA). High purity nitrogen (N_2_) was used as a nebulizing gas at a flow rate of 4 L/minute, and a temperature of 40°C. The gain was set at 8 and 2.0 bar N_2_.

A 125 × 4.0 mm Si-60 column with 5 μm particle diameter (Lichrosher Connecticut, USA) was used. The elution program was a linear gradient with 80:19.5:0.5 (V/V) chloroform: methanol: water: ammonia (NH_3_) at 22 minutes. The column was allowed to equilibrate until the next injection at 27 minutes. The injection volume was 50 μl.

#### Assay of heat shock protein 70 (HSP70)

HSP70 was measured in homogenates of brain cortex and medulla using an ELISA kit, product of Uscn Life Science Inc. Wuhan, China according to the manufacturer’s instructions. The microtiter plate provided in this kit has been pre-coated with an antibody specific for HSP70. Standards or samples are then added to the appropriate microtiter plate wells with a biotin-conjugated polyclonal antibody preparation specific for HSP70. Next, avidin conjugated to HRP is added to each microplate well and incubated. Then, a TMB substrate solution is added to each well. Only those wells that contain HSP70, biotin-conjugated antibody and enzyme-conjugated avidin will exhibit a change in color. The enzyme-substrate reaction is terminated by the addition of a sulfuric acid solution and the color change is measured spectrophotometrically at a wavelength of 450 nm ± 10 nm. The concentration of HSP70 in the samples is then determined by comparing the O.D. of the samples to the standard curve. The minimum detectable level of rat HSP70 detected is less than 0.045 ng/ml.

### Comet DNA assay

Brain tissues were collected from the rat samples, homogenized in 0.075 M NaCl and 0.024 M ethylenediaminetetraacetic acid (EDTA) buffer, pH 7.5, at a ratio of 1 g of tissue to 1 ml of buffer, and then cooled to 4°C. Volumes of 6 μl of brain homogenate were suspended in 100 μl of 0.5% low-melting agarose (LMA) (Sigma-Aldrich, St Louis, MA, USA) and placed onto microscope slides that were cleaned and coated with 300 μl of 0.6% normal melting point agarose (NMP) agarose beforehand. After solidification on ice for 10 minutes, the slides were covered with 0.5% low melting point (LMP) agarose. Once the agarose gel was solidified, the slides were immersed for one hour in an ice-cold lysis solution, consisting of 100 mM Na_2_EDTA, 2.5 M NaCl, 10 mM Tris–HCl, and 1% sodium sarcosinate, which was adjusted to pH 10, using 1% Triton X-100 and 10% dimethyl sulfoxide (DMSO) that were added immediately prior to use. Before electrophoresis, the slides were removed from the lysing solution and placed for 20 minutes in a horizontal electrophoresis unit (near the anode) that was filled with an alkaline buffer to allow the unwinding of DNA and to express alkali-labile damage. The electrophoresis alkaline solution consisted of 1 mM Na_2_EDTA and 300 mM NaOH, pH 13. After the unwinding of DNA, electrophoresis was carried out in the freshly prepared alkaline solution for 20 minutes at 25 V (300 mA). Electrophoresis at high pH resulted in structures resembling comets, as observed by fluorescence microscopy; the intensity of the comet tail relative to the head reflected the number of DNA breaks. Afterwards, the slides were neutralized by adding Tris buffer (pH 7.5), stained with 30 ml of ethidium bromide (Sigma-Aldrich, St Louis, MA, USA) (20 mg/L), and then covered and stored in sealed boxes at 4°C for further analysis.

All preparation steps were performed under dimmed light to prevent additional DNA damage. Images of 100 randomly selected cells (50 counts on each duplicate slide) were analyzed for each sample. For each group, a total of 500 cells were analyzed under a Leitz Orthoplan epifluorescence microscope (magnification 250×) equipped with an excitation filter of 515 to 560 nm and a barrier filter of 590 nm. The microscope was connected through a camera to a computer-based image analysis system (Comet Assay IV software, Perspective Instruments).

Comets were randomly captured at a constant depth of the gel, avoiding the edges of the gel, occasional dead cells, and superimposed comets. DNA damage was measured as tail length (TL = distance of DNA migration from the center of the body of the nuclear core), and tail intensity DNA (TI = % of genomic DNA that migrated during the electrophoresis from the nuclear core to the tail). By presenting all three parameters together, more information could be obtained on the extent of DNA damage.

### Statistical analysis

The data were analyzed using the statistical package for the social sciences (SPSS, Chicago, IL, USA). The results were expressed as mean ± standard error of the mean (SEM). All statistical comparisons between the control and PA-treated rat groups were performed using the one-way analysis of variance (ANOVA) test complemented with the Dunnett test for multiple comparisons. Significance was assigned at the level of *P* <0.05. Receiver operating characteristics curve (ROC) analysis was performed. Area under the curve (AUC), cutoff values, and degree of specificity and sensitivity were calculated.

## Results

Results are presented as mean ± SEM and percentage change of at least six independent measurements. Table 1 presents the mean ± SEM of the GSH (μg/ml), MD (μmoles/ml) and vitamin C (μg/ml) concentrations, and catalase (U/dl) and GPX (U/100 mg) activities in the brain homogenates of the two groups of rats. Compared to control groups, the PA-treated rats exhibited a significant increase in MD, with a concomitant decrease of catalase, GSH, Vitamin C, and GPX (*P* <0.05).

**Table 1:** Mean ± SEM of GSH (μg/ml), MD (μmoles/ml), vitamin C (μg/ml) concentrations and catalase (U/dl) and GPX (U/100 mg) activities in the brain homogenates of the two groups of rats

**Parameters**	**Groups**	**Min.**	**Max.**	**Mean ± SEM**	***P*value**
**GSH (μg/ml)**	Control	49.81	76.8	60.2 ± 3.05	0.05
	PA	22.04	67.96	44.84 ± 4.81	
**GPX**	Control	4.05	6.28	5.17 ± 0.3	0.01
	PA	2.31	2.88	2.49 ± 0.11	
**MD (μmoles/ml)**	Control	0.04	0.11	0.06 ± 0.007	0.001
	PA	0.08	0.42	0.18 ± 0.03	
**Vitamin C (μg/ml)**	Control	15.04	23.89	20.51 ± 1.31	0.011
	PA	6.57	18.88	12.7 ± 1.45	
**Catalase (U/dl)**	Control	3.08	6.08	4.42 ± 0.33	<0.001
	PA	2.03	8.7	3.86 ± 0.84	

*GPX* glutathione peroxidase; *GSH* glutathione; *MD* malondialdehyde; *max* maximum; *min* minimum; *SEM* standard error of the mean.

Table 2 shows a significant elevation of CK (*P* <0.001) activity and a remarkable decrease in brain LDH activity level in the PA-treated group compared to controls.

**Table 2:** Mean ± SEM of brain CK (U/L), and LDH (μmoles/L) concentrations in the two groups of rats

**Parameters**	**Groups**	**Min.**	**Max.**	**Mean ± SEM**	***P*value**
**CK**	Control	44.85	84.72	64.79 ± 5.14	
	PA	164.47	453.44	294.31 ± 36.44	<0.001
**LDH**	Control	1789.25	2509.44	2126.18 ±108.05	
	PA	1370.62	1983.6	1519.76 ± 83.9	

*CK* creatine kinase; *LDH* lactate dehydrogenase; *max* maximum; *min* minimum; *PA* propionic acid; *SEM* standard error of the mean.

Table 3 demonstrates that while PA and acetic acid were significantly elevated (400.55% and 951% increases, respectively), the long-chain polyunsaturated fatty acids (LC-PUFA) were significantly decreased in the brain homogenates of the PA-treated rats compared to those of the untreated controls (*P* <0.05). Docosahexanoic acid (DHA), docosapentaenoic (clupanodonic), eicosapentaenoic acid (EPA), arachidonic acid (AA), and docosapentaenoic (Osbond), linolenic and γ-linolenic acids had the most significantly reduced levels recorded showing percentage decreases of 59.8, 54.95, 56.62, 38.11, 31.02, 25.22 and 16.85%, respectively.

**Table 3:** **Mean ± SEM and percentage change of selective short-chain and long-chain polyunsaturated fatty acids (LC-PUFA) in the brain homogenates of the two groups of rats. Significant level at*p*<0.05**

**Fatty acid**	**Groups**	**Control**	**PA**	***P*value**
**Acetic**	Mean ± SEM	0.02 ± 0.003	0.07 ± 0.007	0.001
	%change	100.00	455.44	
**PA**	Mean ± SEM	0.01 ± 0.003	0.13 ± 0.01	
	%change	100.00	1051.32	
**18:2-n6 Linoleic**	Mean ± SEM	2.64 ± 0.1	1.79 ± 0.1	
	%change	100.00	74.88	
**18:3-n6 γ-linolenic**	Mean ± SEM	1.33 ± 0.06	1.11 ± 0.056	0.065
	%change	100.00	83.15	
**AA**	Mean ± SEM	0.56 ± 0.028	0.35 ± 0.021	0.004
	%change	100.00	61.89	
EPA	Mean ± SEM	0.34 ± 0.04	0.16 ± 0.025	
	%change	100.00	47.38	
**22:5-n3 Docosapentaenoic (clupanodonic)**	Mean ± SEM	1.01 ± 0.07	0.46 ± 0.025	
	%change	100.00	45.05	<0.001
**22:5-n6 Docosapentaenoic (Osbond)**	Mean ± SEM	1.33 ± 0.16	0.93 ± 0.067	
	%change	100.00	69.98	
**DHA**	Mean ± SEM	0.4 ± 0.05	0.16 ± 0.007	
	% change	100.00	40.2	

*AA* arachidonic acid; *DHA* docosahexanoic acid; *EPA* eicosapentaenoic acid; *PA* propionic acid; *SEM* standard error of the mean.

Data presented in Table 4 show clearly that the phospholipids surveyed in the present work (phosphatidylethanolamine (PE), phosphatidylserine (PS), and phosphatidylcholine (PC)) were significantly lower in the brain homogenates of PA-treated rats compared to the untreated control group. Significant level at *P* <0.005

**Table 4:** **Mean ± SEM of PE, PS, and PC levels in the brain homogenates of the two groups of rats. Significant level at*P*<0.005**

**Parameters**	**Groups**	**Min.**	**Max.**	**Mean ± SEM**	***P*value**
**PE**	Control	0.13	0.19	0.17 ± 0.01	
	PA	0.05	0.11	0.09 ± 0.007	
**PS**	Control	0.28	0.35	0.31 ± 0.011	<0.001
	PA	0.06	0.18	0.12 ± 0.018	
**PC**	Control	3.82	4.82	4.46 ± 0.16	
	PA	1.05	2.74	1.97 ± 0.18	

*PA* propionic acid; *PC* phosphatidylcholine; *PE* phosphatidylethanolamine; *PS* phosphatidylserine; *Max* maximum; *Min* minimum; *SEM* standard error of the mean.

Table 5 presents comparison concentrations of caspase3 (pg/ml), IL-6 (pg/ml), TNFα (pg/ml), INFγ (ng/100 mg), and HSP70 (ng/100 mg) in the brain homogenates of the two groups of rats. The data clearly demonstrate the elevation of these parameters in PA-treated rats compared to the untreated control group.

**Table 5:** Caspase3 (pg/ml), IL6 (pg/ml), TNFα (pg/ml), INFγ (ng/100 mg), and HSP70 (ng/100 mg) in the brain homogenates of the two groups of rats

**Parameters**	**Groups**	**Min.**	**Max.**	**Mean ± SEM**	***P*value**
**Caspase3**	Control	110.34	125.50	119.37 ± 2.32	<0.001
	PA	137.97	172.31	154.44 ± 4.3	
**IL-6**	Control	121.33	140.08	129.03 ± 2.85	
	PA	129.34	159.37	143.52 ± 3.36	
**TNF**α	Control	111.01	125.19	119.39 ± 2.16	
	PA	125.73	139.32	131.91 ± 1.55	
**INFγ**	Control	82.47	96.52	90.01 ± 1.82	
	PA	136.37	152.04	141.59 ± 2.22	0.01
**HSP70**	Control	30.28	36.43	32.66 ± 0.89	
	PA	54.28	60.05	57.69 ± 0.91	

*Max* maximum; *Min* minimum; *PA* propionic acid; *SEM* standard error of the mean.

GABA, serotonin, dopamine, adrenaline, and noradrenaline (expressed in ng/10 mg) concentrations were also measured in the brain homogenates of the two groups of rats and results are presented in Table 6. Lower concentrations of the five neurotransmitters in PA- treated rats can be easily observed in the table.

**Table 6:** GABA, serotonin, dopamine, adrenaline, and nor-adrenaline (expressed in ng/10 mg) levels in the brain homogenates of the two groups of rats

**Parameters**	**Groups**	**Min.**	**Max.**	**Mean ± SEM**	***P*value**
**GABA**	Control	95.42	105.03	100.45 ± 1.62	<0.001
	PA	71.58	90.63	79.09 ± 2.05	
**Serotonin**	Control	5.22	8.32	6.99 ± 0.46	
	PA	3.10	5.58	4.17 ± 0.3	
**Dopamine**	Control	15.37	19.98	17.91 ± 0.67	0.007
	PA	6.88	17.63	12.56 ± 1.19	
**Adrenaline**	Control	5.138	6.563	5.98 ± 0.26	
	PA	2.128	3.655	2.83 ± 0.27	<0.01
**Noradrenaline**	Control	2.419	3.575	5.06 ± 0.28	
	PA	4.973	5.113	2.83 ± 0.22	

*GABA* gamma aminobutyric acid; *Max* maximum; *Min* minimum; *PA* propionic acid; *SEM* standard error of the mean.

**Figure 1:**
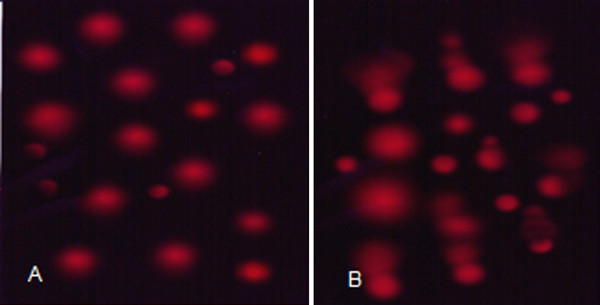
Measure of PA-induced DNA damage by comet assay (A): Control group of rats, (B): PA treated group of rats.

In this study, the DNA damage incurred by treatment with PA is presented in Table 7 and Figure 1 as tailed (%), tail length (μm), and tail moment of the two groups of rats. It can be seen that there was a significant increase in the tail length (5.83 μm increase), tail moment (40 μm increase) and DNA fragmentation in PA-treated rats compared to the untreated controls

**Table 7:** Tailed (%), Tail length (μm), and tail moment of the two groups of rats

**Parameters**	**Groups**	**Min.**	**Max.**	**Mean ± SEM**	***P*value**
**Tailed%**	Control	2%	5%	3.33 ± 0.54	
	PA	16%	20%	18.00 ± 0.71	
**Tail length (μm)**	Control	0.79	1.24	0.97 ± 0.08	<0.01
	PA	6.05	7.52	6.80 ± 0.26	
**Tail moment**	Control	0.64	1.66	0.98 ± 0.21	
	PA	37.264	47.15	41.51 ± 1.8	

*Max* maximum; *Min* minimum; *PA* propionic acid; *SEM* standard error of the mean.

Table 8 demonstrates the ROC analysis for the measured parameters showing AUC, specificity and sensitivity. Of the 32 studied parameters, only 11 parameters (listed in Table 8) show remarkably high sensitivity and specificity (>80%) as measured by ROC analysis. AUC values range from 0.688 to 1.

**Table 8:** ROC analysis of measured parameters in the PA-treated group of rats

**Parameter**	**Area under the curve**	**Cutoff value**	**Sensitivity %**	**Specificity %**
GSH (μg/ml)	0.844	70.12	100.0%	25.0%
GPX (U/100 mg)	1.000	6.03	100.0%	20.0%
MD	0.984	0.09	87.5%	87.5%
Vitamin C levels in brain	0.944	24.23	100.0%	16.7%
Catalase	0.688	5.36	85.7%	12.5%
CK (U/L)	1.000	79.32	100.0%	80.0%
LDH (umoles/L)	0.944	2431.78	100.0%	16.7%
Acetic acid	1.000	0.02	100.0%	40.0%
PA	1.000	0.02	100.0%	80.0%
18:2-n6 (Linoleic)	0.959	2.92	100.0%	14.3%
18:3-n6 (γ -linolenic)	0.847	1.51	100.0%	14.3%
AA	1.000	0.64	100.0%	14.3%
EPA	1.000	0.665	100.0%	100%
22:5-n3 Docosapentaenoic (clupanodonic)	1.000	0.635	100.0%	100%
22:5-n6 Docosapentaenoic (Osbond)	0.959	1.49	100.0%	14.3%
DHA	1.000	0.44	100.0%	14.3%
PE	1.000	0.19	100.0%	25.0%
PS	1.000	0.34	100.0%	25.0%
PC	1.000	4.89	100.0%	25.0%
Caspase3	1.000	125.45	100.0%	75.0%
IL-6	0.917	136.48	66.7%	75.0%
TNFα	1.000	125.03	100.0%	100.0%
INFγ	1.000	95.15	100.0%	80.0%
HSP70	1.000	35.19	100.0%	80.0%
GABA	1.000	93.025	100.0%	100.0%
Serotonin	0.944	8.19	100.0%	100%
Dopamine	0.972	19.69	100.0%	25.0%
Adrenaline	1.000	6.65	100.0%	33.3%
Noradrenaline	1.000	5.13	100.0%	33.3%
Tailed%	1.000	4.70	100.0%	66.7%
Tail length (μm)	1.000	1.18	100.0%	66.7%
Tail moment	1.000	1.51	100.0%	66.7%

*AA* arachidonic acid; *CK* creatine kinase; *DHA* docosahexanoic acid; *EPA* eicosapentaenoic acid; *GABA* gamma aminobutyric acid; *GPX* glutathione peroxidase; *GSH* glutathione; *LDH* lactate dehydrogenase; *MD* malondialdehyde; *PA* propionic acid; *PC* phosphatidylcholine; *PE* phosphatidylethanolamine; *PS* phosphatidylserine; *ROC* receiver operating characteristics.

**Figure 2:**
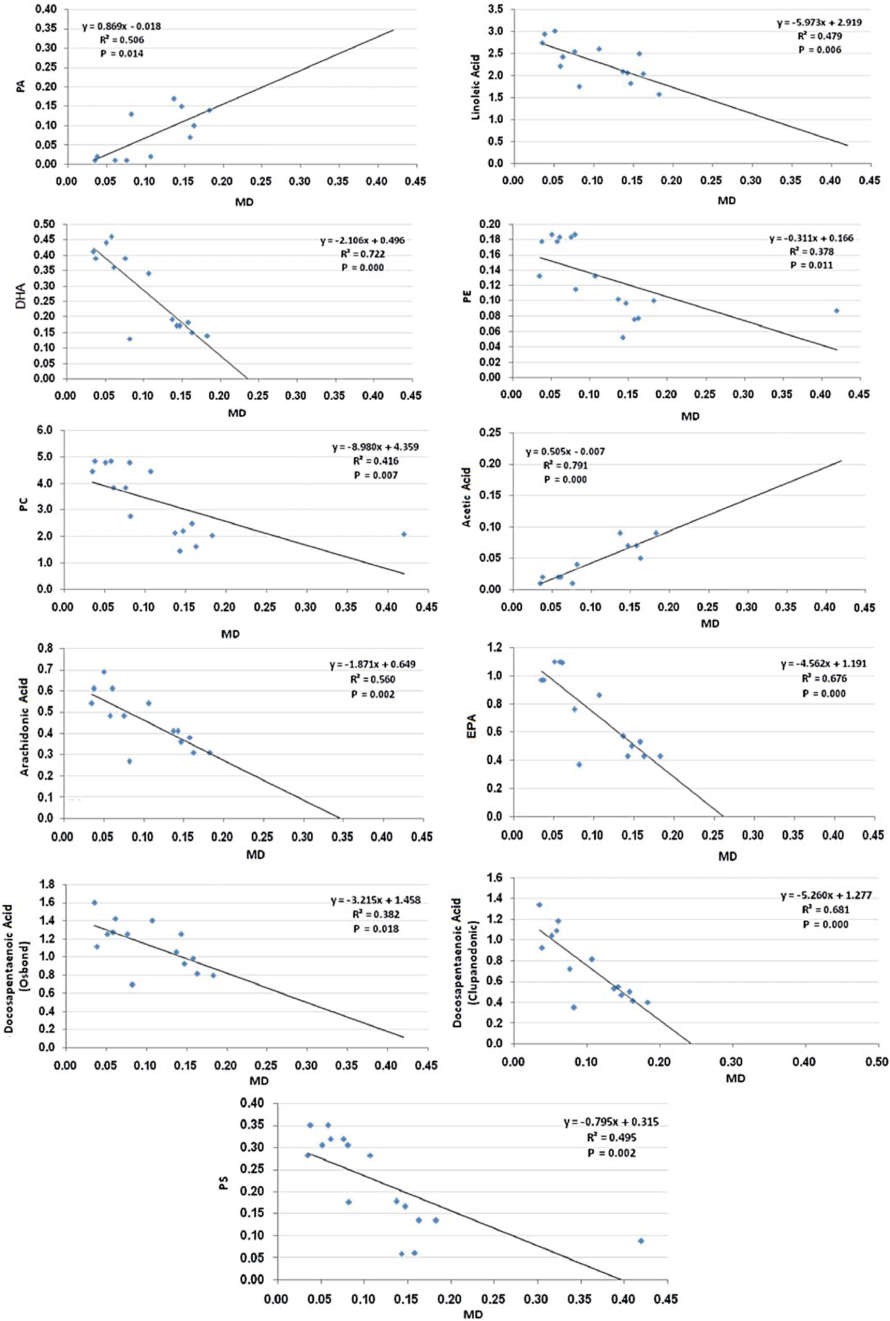
Pearson’s correlations of the most significant positive and negative correlated variables with best fit line curve.

**Figure 3:**
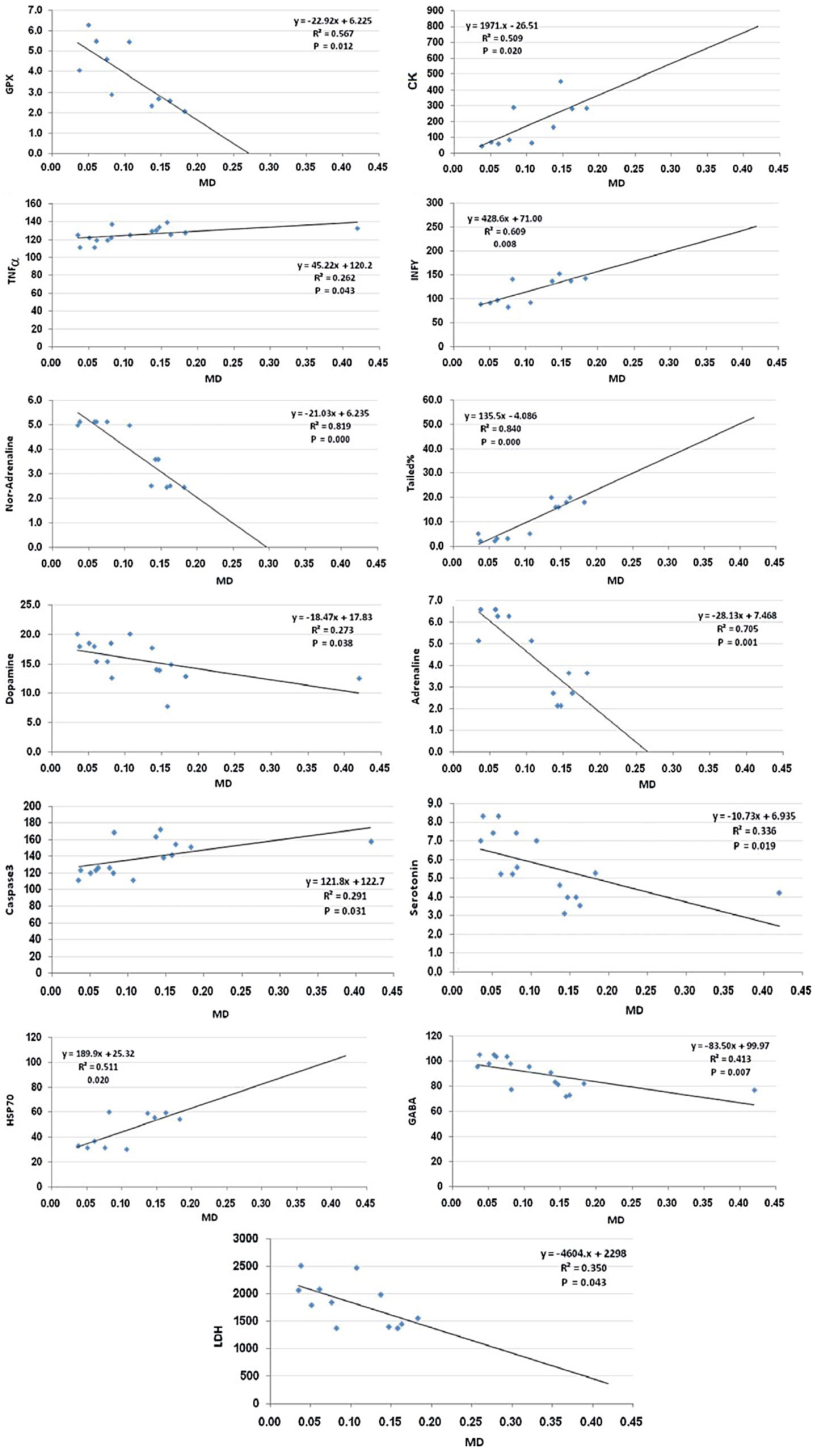
Pearson’s correlations of the most significant positive and negative correlated variables with best fit line curve.

Figures 2 and 3 shows the Pearson’s correlations of the most significant positive and negative correlated variables with best fit line curve. Out of the 402 Pearson’s correlations between the 32 measured parameters, only those representing the significant correlations between MD and the other parameters were selected to be presented.

## Discussion

Several parallels have explicitly been drawn between mechanisms of neurodevelopmental diseases and environmental neurotoxicity in recent research. In fact, the literature provides ample evidence for interrelated pathways that result in neuronal cell death. The challenges for the early intervention and prevention of such debilitating conditions include the subclinical detection of insult in populations at risk and in defining the specific neural targets involved in the process. In this context, experimental studies on animal models can provide valuable data as to the determination of regional and cellular vulnerability to particular neurodevelopmental diseases and the identification and management of clinical biomarkers.

### Oxidative stress markers

The findings of the present study revealed a significant increase in MD, a significant marker of oxidative stress, with a concomitant decrease of catalase, GSH and GPX in the brain homogenates of the PA-treated rats, which corroborated the role of oxidative stress in the etiology of autism (Table 1). This latter finding is in accordance with previously described results reporting that while red-cell lipoxidation is twice as high among autistic children than in age-matched controls, GSH, GPX and catalase are significantly lower [26,27].

Zoroglu et al. 2004 attributed antioxidant decreases to lower production or greater consumption rates, implying greater vulnerability of the autistic brain to oxidants. The findings are also in line with a recent study on brain regions that reported a significant increase of lipid hydroperoxide levels in the cerebellum and temporal cortex of autistic children [28]. Similar results were also recorded for the animal model of MacFabe *et al*. [9] which showed elevated lipid peroxides and lower GSH levels, GPX and GRX activities, together with typical cognitive disability, repetitive behavior, object-directed behavior, and social behavior in rats to which PA was administered intraventricularly [29].

The results also revealed an increase in CK and a decrease in LDH, two enzymes related to energy metabolism (Table 2). These results are in line with the findings of a previous study by Al-Mosalim et al. [30] that reported lower ATP levels in red blood cells together with elevated lactate and CK activities in the plasma of Saudi autistic children compared to age-matched controls. Furthermore, the activation of brain CK observed in the present study could be easily related to the significant increases in the activities of Na^+/^K^+^ ATPase and Ca^2+^/Mg^2+^ ATPase and the marked decrease in the expression of mitochondrial electron transport chain (ETC) complexes in different regions of the brain of autistic subjects compared with their age-matched controls and suggests the contribution of these enzymes to the abnormal energy circuit functioning in autism [28].

### Lipid profile markers

There is growing interest in recent research on the potential roles of the n-3 polyunsaturated fatty acid (PUFA) docosahexanoic acid (DHA) and precursor eicosapentaenoic acid (EPA) with regard to brain structure, function, and mental health in human beings [31-33]. DHA is the most abundant PUFA in brain membrane phospholipids, which is indicative of its role in membrane fluidity and associated metabolic and neural activities. In fact, DHA is particularly concentrated at neural synapses, sites of neurotransmitter signaling. Omega-6 PUFA arachidonic acid (AA) is also abundant in the brain, reflecting a key role in brain structure and function. Likewise, the AA precursor, gamma-linolenic acid (γLA), and n-3 DHA precursor EPA are all considered to play key roles in brain functioning especially via the synthesis of eicosanoids that have anti-inflammatory, anti-thrombotic, and vasodilatory properties [33]. As far as the present study is concerned, the findings indicated similar correlations between the levels of PA and acetic acids as short chain fatty acids and long chain (LC)-PUFA in the brain homogenates of both the treated and untreated groups of rats (Table 3). It could be easily observed that while PA and acetic acids were significantly elevated, the LC-PUFA were significantly decreased in the brain homogenates of the PA-treated rats compared to those of the untreated controls. Based on this observation, the depletion recorded for most LC-PUFA levels could presumably be attributed to the brain dysfunction in the autistic patients and suggests that dietary supplementation with LC-PUFA might assist in the management of the childhood behavioral and learning difficulties related to autism.

Furthermore, and considering the fact that the brain is often reported to be capable of synthesizing only a few fatty acids and that most fatty acids must, accordingly, pass through the blood into the brain [34], the data obtained in this respect could be easily linked to autism. In fact, El-Ansary et al. [16] previously provided plausible links that related the occurrence of lower PA in the plasma of autistic patients to elevated levels of PA in their brain. They attributed the lower plasma PA to the high rate of influx from blood to brain. In fact, and compared to other fatty acids, propionate was previously reported to cross the BBB with a brain uptake index of 43.53 and a low Km value of 2.03 [15]. Since the lower the Km, the higher the affinity of the transporters for the substrates, then an uptake index of 43.53% and a Km value of 2.03 are enough to facilitate the penetration of propionate into the brain cell, which could explain the elevation of PA in the brain homogenates of the treated rats. In fact, the data obtained from the intraventricular administration of PA to rats, such as those presented in the animal model of MacFabe et al. [9], could provide the means to model a number of aspects pertaining to human ASD in rats. Data generated from animal model studies in which PA is orally administered might, on the other hand, provide evidence for the importance of the gut-to-brain pathway in the etiopathology of autism and open new opportunities for the development of feasible pharmaceutical and/or nutritional approaches for the treatment and prevention of autism.

Phospholipids enriched in unsaturated fatty acids (phosphatidylethanolamine (PE), phosphatidylserine (PS), and phosphatidylcholine (PC)) have often been reported to be essential for the normal neurological function of the brain. In fact, neurodegeneration was previously attributed to abnormal metabolism of phospholipids in the brain [35]. The composition of erythrocyte membrane phospholipid has also been shown to correlate with brain phospholipid composition [36] and to be a potentially useful marker for neurological diseases [37,38]. The phospholipids surveyed in the present work, namely PE, PS, and PC, were significantly lower in the brain homogenates of PA-treated rats than in those of the untreated control group (Table 4). This could be attributed to a number of autistic features particularly those related to oxidative stress and inflammatory responses, two mechanisms that have often been reported to play critical roles in the pathophysiology of autism. This result is also in line with the findings previously reported by Pandey et al. [39] showing that omega-6 phospholipids, such as PC, exhibited anti-inflammatory properties through the inhibition of TNF*α* and H_2_O_2_-induced mitogen-activated protein kinase (MAPK) in the neuronal cell line SH-SY5Y and prevented the phosphorylation and activation of nuclear factor-kappa B (NF-kappaB). The significant depletion of brain PE, PC, and PS in the brain homogenates of PA-treated rats is also in agreement with the findings recently reported by El-Ansary et al. [40] who described a similar depletion in terms of those phospholipids in the plasma of autistic patients compared to age-matched controls. These depletions of brain phospholipids, as well as the implied contribution of PA neurotoxicity in the etiopathology of autism, are also consistent with the hypothesis previously advanced by Brown and Austin [41] that proposed dysregulated phospholipid metabolism as the underlying biological component of autism.

### Neuroinflammation related markers

Several reports in the literature suggest that a combination of environmental and, possibly, *in utero* and autoimmune risk factors or CNS localized inflammation may contribute to the pathogenesis of ASD [42-44]. The findings of the present study clearly demonstrate the elevation of IL-6, TNFα, and IFNγ in the brain homogenates of PA-treated rats (Table 5). This could provide support for the contribution of PA neurotoxicity, as an environmental factor and metabolic product of enteric bacteria, to the pathogenesis of autism, which is in line with the findings reported in several clinical studies related to autistic brain cytokines. Li et al. [45] has, for instance, developed a flow cytometry method (multiplexed bead analysis) to measure cytokine levels in brain (cerebral cortex) extracts. They showed that proinflammatory cytokines (TNFα, IL-6, and granulocyte macrophage colony-stimulating factor), Th1 cytokine (IFNγ), and chemokine (IL-8) were significantly increased in the brains of ASD patients compared to those of controls which could, presumably, suggest a conserved function of PA in the etiology of autistic features. A recent study by Ashood et al. [46] reported that, compared to age-matched typically developing children and children with developmental disabilities other than autism, autistic children were noted to undergo increases in the cytokine levels that were associated with more impaired communication and aberrant behaviors. Likewise, the neuroinflammation recorded for the group of rats to which PA was orally administered is consistent with the findings of a recent study by MacFabe et al. [29] which showed, through the immunohistochemical analysis of brain tissues from intracerebroventricularly PA-injected rats, a remarkable reactive astrogliosis and activated microglia, thus providing evidence for an innate neuroinflammatory response.

### Neurotransmission related markers

Multiple lines of evidence have suggested that the GABAergic system is disrupted in the brains of individuals with autism and that altered inhibition within the network is likely to influence the ability to perceive emotional expressions [47]. In fact, the findings of the present work revealed marked decreases in the GABA, serotonin, and dopamine levels in the brain homogenates of PA-treated rats. The decrease recorded for brain serotonin and dopamine is, indeed, in disagreement with a previous study by Narita et al. [48] which reported an increase in the hippocampal serotonin and frontal cortex dopamine levels of rats to which thalidomide and valproic acid were administered as two potential autism-inducing teratogens. This discrepancy could be attributed to age-related differences. In fact, while the animals in the work of Narita et al. [48] were given those two teratogens on embryonic days (E)2, E4, E7, E9, and E11, the present study experimented with 21-day-old rat pups. Similar decreases were previously reported in several imaging studies on serotonin transporter binding or tryptophan retention in autistic patients, such as the work of Azmitia et al. [49] which reported on a decrease in the brain serotonin system of autistic patients and provided immunocytochemical evidence for an increase in the 5-hydroxytryptophan (5-HT) axons (immunoreactive to 5-HT transporter) of postmortem brain tissues taken from 2.8- to 29-year-old autistic donors compared to healthy controls. In autistic donors eight years of age and older, several types of dystrophic 5-HT axons were detected in the termination fields [50]. Winter et al. [51] have also reported a similar decrease in serotonin and dopamine following prenatal viral infection, potentially modeling disruptions that occur in patients with schizophrenia and autism.

The decrease recorded in the present study with regard to dopamine is also consistent with the earlier study of Garnier et al. [52] which registered elevated dopamine hydroxylase and homovanillic acid (HVA) in autistic children and reported the involvement of dopamine dysfunction in the production of autistic symptoms. Finally, the observed reduction in adrenaline and noradrenaline is consistent with the recorded decrease in dopamine, as a vital neurotransmitter for normal functions in the brain and serves as a precursor of both [53].

### Pro-apoptosis related markers

Table 7 demonstrates elevated levels of caspase-3, a pro-apoptotic marker, and HSP70 as a stress-induced protein in brain homogenates of PA-treated rats compared to controls. A similar increase in caspase-3 has recently been reported by Olczak et al. [54] who provided evidence for an increase of caspase-3 in developing rat brains as neurotoxic effects of thimerosal, a mercurial compound involved in the etiopathology of autism. This rise could be attributed to mitochondrial dysfunction, disruption of the BBB, and/or apoptosis, which are neurotoxic markers of PA. Stao et al. [55] reported that BBB disruption is an early event that is often followed by increased HSP70 expression and apoptosis. They speculate that 3-nitropropionic acid damages endothelial cells, leading to vasogenic edema and apoptosis. The findings of the present work seem to corroborate such speculation, giving evidence for a highly significant increase in the tail length, tail moment (comet DNA assay), and DNA fragmentation in PA-treated rats compared to the untreated controls (Table 7 and Figure 1).

Of the 402 Pearson’s correlations between the 32 measured parameters, only those representing the significant correlations between MD as a marker of oxidative stress, a major mechanism of PA neurotoxicity, and the other parameters are shown in Figures 2 and 3. It can be easily noticed that there are positive associations between brain MD and short chain fatty acids (PA and acetic acid), proinflammatory markers (TNFα, INFγ, IL-6, and HSP70) and pro-apoptotic markers (caspase3, DNA tail length and moment). Negative correlations were observed between MD and GPX, GSH, long chain fatty acids and neurotransmitters (GABA, 5-HT, dopamine, adrenaline and nor-adrenaline). This may suggest that increased oxidative stress, a status recently reported to exert both etiologic and clinical significance in autism, [56] is the most important mechanism of PA neurotoxicity.

Of the 32 studied parameters, only 11 show remarkably high sensitivity and specificity (>80%) as measured by ROC analysis (data not shown). AUC values of (0.688 to 1) are statistically satisfactory to suggest these parameters (listed in Table 8) as markers of PA neurotoxicity.

Overall, the suggested etiology of autistic features in rats orally administered PA, as were the subjects of the present study could find support in the most recent study of Ossenkopp et al. [57] which showed that 500 mg/kg intraperitoneal administration of PA could produce both conditioned taste avoidance and conditioned place avoidance in rats as two autistic behaviors. Further, it provided ample evidence for the conserved neurotoxic effects of PA during the development of rat brains, thus suggesting that this enteric bacterial toxin and commercialized food additive can be considered as a significant epigenetic component that may be contributing to the alarming rates with which autism is increasing. Moreover, the orally administered PA used in the present study could point to connections between the gut-to-brain axis and the pathogenesis of autism and, in turn, to the potential promising opportunities that those links may offer for pharmaceutical and/or nutritional approaches for the treatment and prevention of autism.

## Abbreviations

AA, Arachidonic acid; ASDs, Autism spectrum disorders; AUC, Area under the curve; BBB, Blood–brain barriers; CK, Creatine kinase; CNS, Central nervous system; DHA, Docosahexanoic acid; EPA, Eicosapentaenoic acid; FFG, Fusiform gyrus; GABA, Gamma amino-butyric acid; GPX, Glutathione peroxidase; GSH, Glutathione; H2O2, Hydrogen peroxide; HSP70, Heat S-hock protein70; INFγ, Interferon-γ; IL6, Interlukin-6; LC-PUFA, Long chain polyunsaturated fatty acid; LDH, Lactate dehydrogenase; MD, Malondialdehyde; ROC, Receiver operating characteristics curve; PC, Phosphatidylcholine; PE, Phosphatidylethanolamine; PS, Phosphatidylserine; PCC, Posterior cingulate cortex; PUFA, Polyunsaturated fatty acid; PA, Propionic acid; TNFα, Tumor necrosis factor α.

## Competing interests

The authors declare that they have no competing interest.

## Authors’ contributions

AE designed the study and drafted the manuscript. ABB helped to draft the manuscript and performed the statistical analysis. MO helped with the English polishing and participated in the design of the study. All authors have read and approved the final manuscript.
